# Selected stem cell populations in pediatric acute lymphoblastic leukemia

**DOI:** 10.3389/fimmu.2024.1446687

**Published:** 2024-09-25

**Authors:** Anna Krętowska-Grunwald, Małgorzata Sawicka-Żukowska, Aleksandra Starosz, Maryna Krawczuk-Rybak, Marcin Moniuszko, Kamil Grubczak

**Affiliations:** ^1^ Department of Regenerative Medicine and Immune Regulation, Medical University of Bialystok, Bialystok, Poland; ^2^ Department of Pediatric Oncology and Hematology, Medical University of Bialystok, Bialystok, Poland; ^3^ Clinical Department of Allergic and Internal Diseases, Medical University of Bialystok, Bialystok, Poland

**Keywords:** acute lymphoblastic leukemia, very small embryonic-like stem cells, hematopoietic stem cells, stromal cell-derived factor 1, regenerative medicine

## Abstract

**Introduction:**

Acute lymphoblastic leukemia is characterized by a disturbed maturation of hematopoietic stem cells (HSCs) resulting in development of a malignant clone. Despite relatively positive outcome, there are still instances of disease relapse occurring due to ineffective disease eradication or primary leukemic clone alterations. Unclear significance of stem cells in the course of ALL led us to investigate and establish crucial changes in two stem cell populations - very small embryonic-like stem cells (VSELs) and HSCs during the induction phase of treatment.

**Methods:**

In a retrospective study selected stem cells in peripheral blood and bone marrow of 60 pediatric ALL subjects and 48 healthy controls were subjected to flow cytometric analysis at 4 different time points.

**Results:**

Both VSELs and HSCs were elevated at the moment of ALL diagnosis compared to healthy controls, but profoundly decline until day 15. Further observations revealed an increase in HSCs with a concomitant depletion of VSELs until week 12. ALL patients with high HSCs showed positive correlation with bone marrow blasts at diagnosis. Patients with lower VSELs or HSCs at diagnosis had slightly improved response to applied therapy. We observed higher initial bone marrow lymphoblast values in patients with lower VSELs or higher HSCs in the high-risk group. The significance of VSELs in predicting treatment outcome can be illustrated by lower day 15 MRD level of patients with lower VSELs at diagnosis.

**Discussion:**

We found HSCs and VSELs to be valid participants in pediatric ALL with possible contribution in the neoplastic process and prediction of initial treatment outcome.

## Graphical Abstract

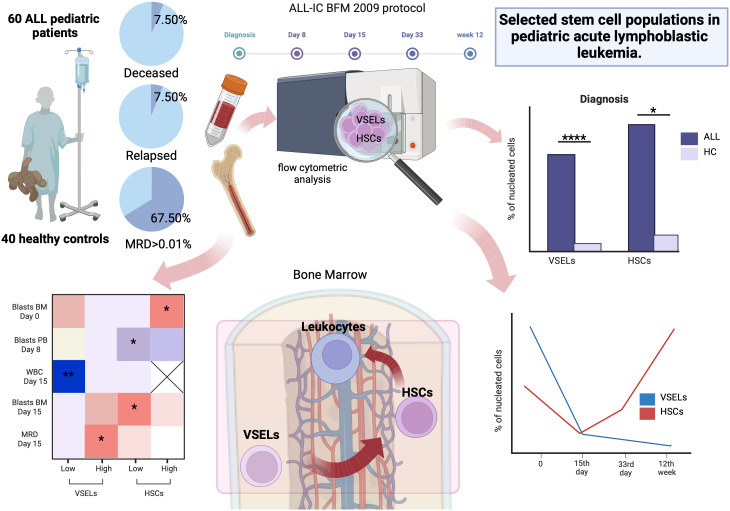

## 1 Introduction

Acute lymphoblastic leukemia (ALL) represents a malignant proliferation of lymphoid cells with their development arrested during early maturation in a process defined as leukemogenesis (1). Lymphoblasts derived from the B-cell lymphoid lineage are the dominant population in ALL. The occurrence of pediatric acute lymphoblastic leukemia is found to be age-related, with peak incidence between 1 to 4 years (2, 3). Udroiu et al. attributes this reliance to interleukin 7-independent B-cell development occurring until the age of 5 and after the initial genetic mutation created a preleukemic clone *in utero* (4).

The development of ALL involves the interception of normal HSC maturation by several “hits” of chromosomal translocations and genetic mutations; the first initiating the generation of “leukemic stem cells” and the latter turning them malignant (5). These alterations change lymphocyte precursors into a clone which is resistant to programmed cell death, lacks differentiation ability and exhibits independency to bone marrow stromal cells (6). Lymphoblasts occupying the bone marrow niche of quiescent hematopoietic stem cells acquire this dormant characteristic and can be the culprit of therapy resistance (7). On the other hand Yamashita et al. state that although treatment-resistant leukemic stem cells were initially identified as the cause of post-therapy relapse in acute myeloid leukemia patients, more advanced analysis assign this role to mutations in preleukemic HSC clones (1).

Hematopoietic stem cells (HSCs) are tissue-bound progenitors which reside in a quiescent state in the endosteum of the bone marrow and can become activated under stress-induced conditions such as chemotherapy, transforming them into a proliferating state (8–10). Nearly 10% of all HSCs can be found in the peripheral blood of adult human subsets, with no pediatric values clearly stated (11). In accordance with results of Georgievski et al., leukemic cells seem to interfere with HSCs development through the creation of extracellular vesicles (EV) in the bone marrow niche. These EV were found to carry a disturbed metabolite load, i.e. increased cholesterol, which accelerated the metabolism of hematopoietic stem and progenitor cells leading to their exhaustion (12). In the study of Baird et al., chemotherapy did not seem to exert a significant effect on those HSCs which remained quiescent in the bone marrow, confirmed by spontaneous increase in the number of blood cells after their initial reduction with chemotherapy (13). Clapes et al. identify chemotherapy as a stress agent which stimulates HSC proliferation out of their quiescence through melanoma differentiation-associated protein 5 (MDA5), and enables their differentiation into cells reconstituting the hematopoietic pool (10). A recent study by Tang et al. indicated that chemotherapy does in fact impact the quiescent state of HSCs, promoting their entry into the cell cycle. Chemotherapy induced apoptosis of the bone marrow HSCs and impaired their reconstitution capability after transplantation in B-ALL murine models (14). This may be the cause of chemotherapy-induced residual injury of the bone marrow, leavin*g* the patients with long-lasting hematological disorders (15).

The last two decades brought novel insight into the topic of HSC precursors. Reports on the subject of very small embryonic-like stem cells (VSELs) date back to 2006 when they were first described in *Leukemia* journal as Sca-1+lin-CD45- in a murine model and later as lin-CD45-CD133+ in humans, isolated from various tissue compartments (bone marrow, cord or peripheral blood, etc.) (16–18). Given their pluripotency and hence the ability to differentiate into the three primordial germ layers both, *in vitro* and *in vivo*, they became the topic of investigation in regenerative medicine, with a crucial role in the course of human development and growth (19–22). The bone marrow reserve of VSELs of healthy adults is quite scarce constituting around 0.01-0.001% of nucleated cells (23). Interestingly their number seems to increase in the bone marrow and peripheral blood of individuals undergoing stress conditions, including: myocardial infarction, inflammatory diseases, cancer, skin damage (24–27). To date, VSELs themselves were not shown to exhibit any hematopoietic activity in both *in vitro* and *in vivo* settings. They gain this ability and differentiate into HSCs upon coculture with OP9 stroma cell line (17, 23). However, the direct association between VSELs and HSCs remained ambiguous until recently Ratajczak et al. proposed a Lin-Sca-1+CD45+CD38+c-Kit-H-2 Kb+ cell population to be the linking factor explaining the enrichment of VSELs with HSCs (28). This has confirmed the long-stated hypothesis of VSELs serving as a contingency reservoir for lineage-restricted cells such as HSCs (23, 29). According to Domingues et al. VSELs serve as precursors of cells exhibiting vasculogenic endothelial potential (30). Noteworthy, these stem cells do not complete the development of blastocyst and, concomitantly, do not form teratomas as presented in the context of iPCS or ECS (31, 32).

Influence of VSELs on the course of acute lymphoblastic leukemia remains an undiscovered area. Importantly, recent studies indicate a substantial link between these cells and HSCs, which demonstrate a substantial role in leukemogenesis. The presented study is the first to our knowledge evaluating the association between VSELs and HSCs in the course of ALL. Moreover, we investigated the effects of administered pediatric ALL treatment on these stem cells, additionally evaluating their prognostic value in the therapy outcome estimation.

## 2 Materials and methods

### 2.1 Patients’ characteristics

In the study a total number of 60 acute lymphoblastic leukemia pediatric patients was enrolled from 2013 to 2017 at the Department of Pediatric Oncology and Hematology, Medical University of Bialystok, Poland. Control group of 48 healthy subjects consisted of patients with excluded autoimmune, oncological, and inflammatory conditions, selected in the course of routine diagnostic procedures. Studied groups demonstrated consistency in the context of sex distribution and age. Complete characteristics of the studied subjects are included within the supplementary materials (**Supplementary Figure 1**). Bone marrow and peripheral blood samples were collected during standard diagnostic procedures conducted at the clinical department. Experimental material from the bone marrow was collected during induction therapy: at the moment of diagnosis, on day 15, 33 and 12-week point of the patients’ management and from the peripheral blood: at the moment of diagnosis on day 8, 15 and 33. Written informed consent was obtained from all patients (parent or legal guardian for underage subjects). The study protocol was approved by the Ethical Committee at the Medical University of Bialystok – approval number: APK.002.603.2021. Treatment of the patients was performed in accordance with ALL-IC BFM 2009 protocol. The Induction Therapy (Protocol I’A/IA) involved the use of prednisone, vincristine, daunorubicin, L-asparaginase and intrathecal methotrexate. MRD (minimal residual disease) values were evaluated on day 15^th^, and together with clinical and laboratory data were the factors for the disease risk-groups stratification.

### 2.2 Flow cytometric evaluation of selected stem cells

Immunostaining with fluorochrome-conjugated monoclonal antibodies was performed on 600μl of tested subjects’ bone marrow. Ex vivo assessment of the samples implemented the following antibodies: anti-Lineage1 FITC (anti-CD3, clone SK7; anti-CD14, clone MφP9; anti-CD16, clone 3G8; anti-CD19, clone SJ25C1; anti-CD20, clone L27; anti-CD56, clone NCAM16.2), anti-CD235a FITC (clone GA-R2), anti-CD45 PE (clone HI30) (BD Bioscience), anti-CD133 APC (clone AC133) (Miltenyi Biotec). Samples were incubated for 25 minutes in the dark at room temperature. Subsequent washing out of unbound antibodies after incubation was followed by cell fixation with the use of CellFix reagent (BD Bioscience) and samples stored at 4°C. FACS Calibur flow cytometer (BD Bioscience, San Jose, CA, USA) was used for data acquisition. Flow cytometric data were analyzed with FlowJo software (Tree Star Inc., Ashland, OR, USA). Initially, small-sized cells of 2-6μm were gated using relative size parameter (forward scatter, FSC) supported by size beads (Thermo Fisher), and granularity/complexity (side scatter, SSC) properties. Next, bidirectional gating strategy was applied for technical verification of the rare events analysis. Thus, Lineage-positive cells were excluded and then CD133+CD45+ and CD133+CD45- populations determined. On the other hand, Lineage-CD133+ were gated followed by detection of CD45+ and CD45- cells. Besides morphology, studied phenotypes of stem cells included: Lineage-CD133+CD45+ (HSC) and Lineage-CD133+CD45- (VSEL) (22, 31–33). Gating strategy implemented in the study has been presented within supplementary material (**Supplementary Figure 2**).

### 2.3 Immunoenzymatic assessment of the SDF-1

Supernatants of the ALL patients’ and control group bone marrow was used for the evaluation of the SDF-1/CXCL12 (stromal cell-derived factor 1) concentration. SDF-1 levels were established using immunoenzymatic technique, in accordance with the protocol included within the Human CXCL12/SDF-1 ELISa kit (R&D Systems). Protein concentration related absorbance was measured with the use of LEDETECT96 microplate reader (Labexim) as 450nm wavelength with correction applied.

### 2.4 Biostatistical data processing

Collected data were analyzed with the use of GraphPad Prism 9.0 statistical software (GraphPad Prism Inc., San Diego, CA, USA). Due to non-Gaussian distribution, non-parametric Mann-Whitney U test was implemented to compare differences between studied groups. Differences between time points in the course of therapy were analyzed with application of ANOVA tests (with Fishers’ LSD post-test). Correlation analysis between selected parameters was performed using Spearmen test, with exact coefficient values (R values) presented on the graphs. Fishers’ exact test was applied for relative risk assessment, and relative values with 95% confidence interval showed on the graphs. Statistical significance level was set at p value of 0.05, and demonstrated on the graphs with asterisks or exact p values: * - p < 0.05, ** - p < 0.01, *** - p < 0.001, **** - p < 0.0001.

## 3 Results

### 3.1 Differences in selected bone marrow stem cell populations distribution between ALL and control group subjects at admission

**Figure 1:**
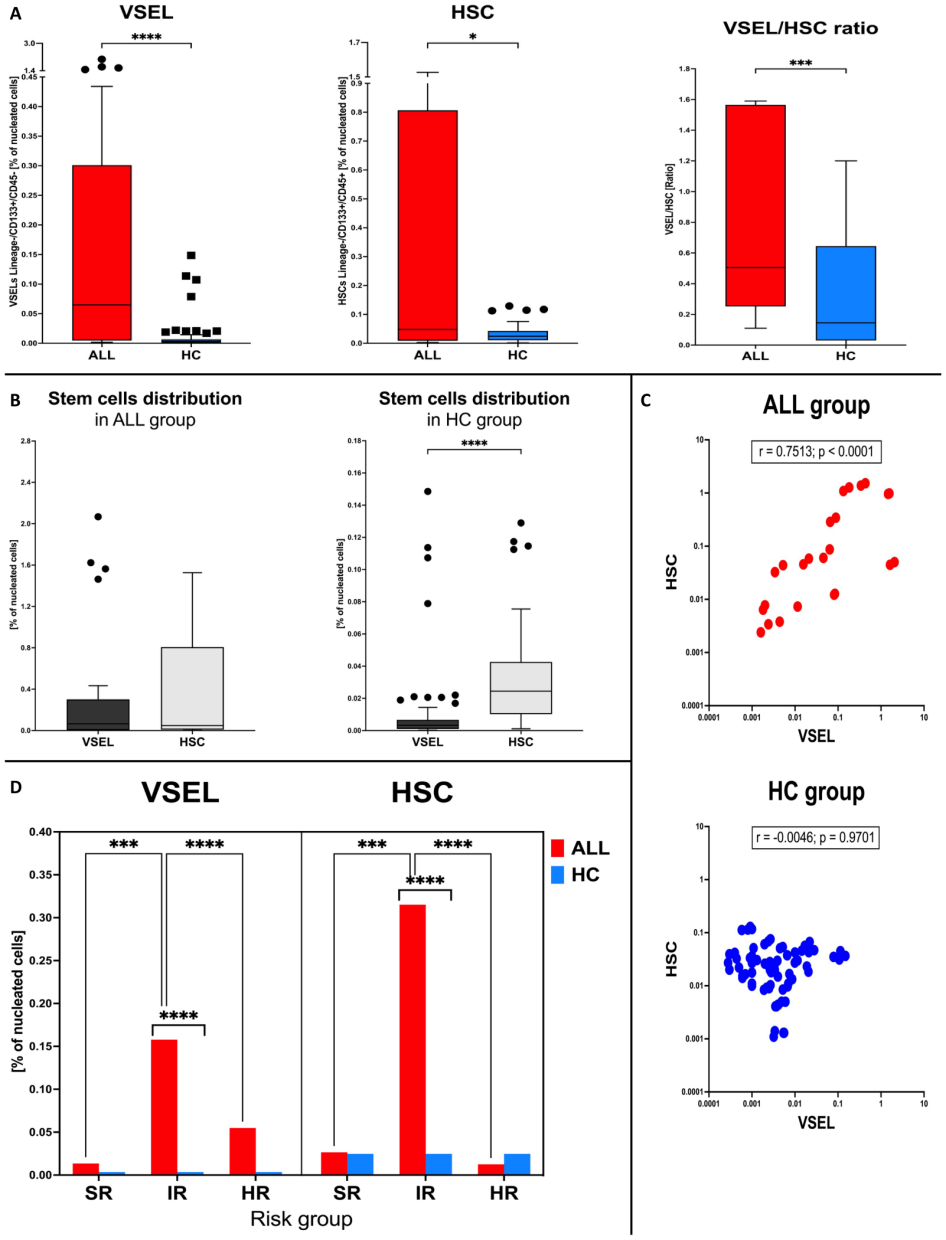
Investigation of VSEL and HSC populations in bone marrow of ALL patients at time of admission. Changes in VSEL, HSC and their ratio between ALL patients (ALL) and the healthy control group (HC) **(A)**. Variations in mutual distribution of VSEL and HSC in the studied groups **(B)**. Correlation between VSEL and HSC in patients with ALL and control group **(C)**. ALL risk-group stratification of patients (SR, standard; IR, intermediate; HR, high risk) for assessment of differences in reference to the control group, in context of VSEL and HSC levels **(D)**. Data presented as Turkey boxplots or median values exclusively; correlation graphs with coefficient and p values. Statistically significant values were indicated with asterisks: * - p < 0.05, *** - p < 0.001, **** - p < 0.0001.

Initial analysis of selected stem cells in the bone marrow of studied subjects revealed significantly higher frequency of VSELs (p < 0.0001) and HSCs (p = 0.0267) in ALL pediatric patients compared to the healthy control group. These changes were most pronounced in the context of VSELs additionally resulting in higher VSEL/HSC ratio in the leukemic subjects (p = 0.0006) (**Figure 1A**). An in-depth examination of stem cells relations revealed that despite significantly lower VSELs compared to HSCs in the control group (p < 0.0001), the difference is diminished in ALL patients (**Figure 1B**). Furthermore, those disease-affected subjects exclusively demonstrated a significant strong positive correlation between VSEL and HSC populations (r = 0.7513, p < 0.0001), with no such association in control group (r = -0.0046, p = 0.9701) (**Figure 1C**).

Stratification of ALL patients into risk groups allowed us to verify VSELs and HSCs levels within standard-risk (SR), intermediate-risk (IR) and high-risk (HR) subgroups. We showed that above-described higher values of the studied stem cell populations are linked to the IR and HR patients predominantly. In addition, most pronounced changes were found in the IR group of ALL patients compared to two other subgroups. Noteworthy, those subjects showed highly significant differences compared to the healthy control group in the context of both, VSEL (p < 0.0001) and HSC (p <0.0001) populations (**Figure 1D**).

### 3.2 Therapy effects on the bone marrow VSEL and HSC populations in ALL pediatric patients

**Figure 2:**
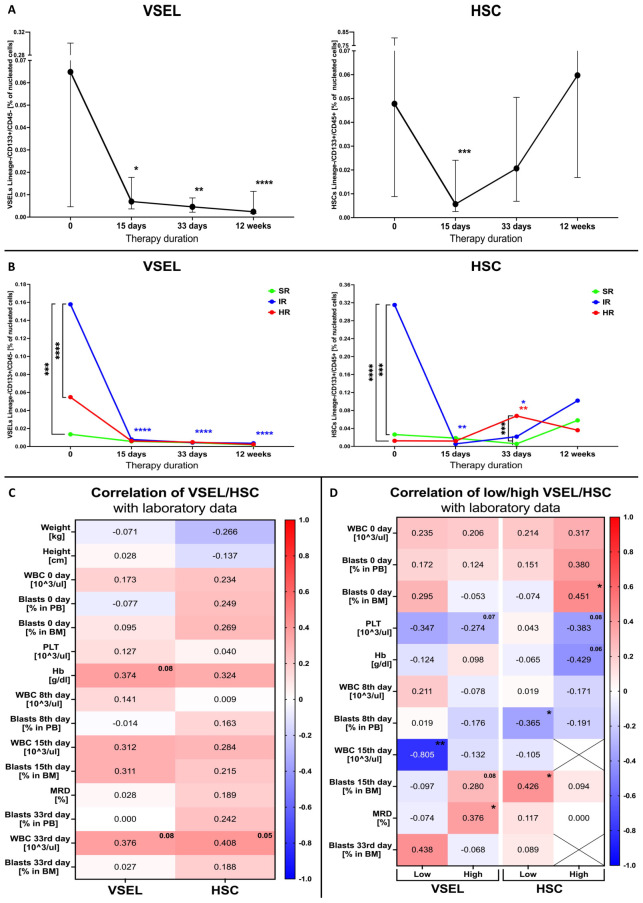
Therapy effects on VSEL and HSC populations in the bone marrow in ALL pediatric patients. Changes in VSEL and HSC in the course of therapy **(A)**. Analysis of differences in stem cell response to therapy in ALL patients distinguished by risk-based stratification (SR, standard; IR, intermediate; HR, high risk) **(B)**. Correlation of VSEL or HSC populations (reported at time of admission) with diagnostic parameters in the course of therapy **(C)**. Differences in mutual associations between selected stem cells and laboratory data in median-based low/high VSEL or HSC patient groups **(D)**. Data presented as medians with interquartile range or median values exclusively; heat-maps demonstrating correlation coefficient values. Statistically significant differences were indicated with asterisks: * - p < 0.05, ** - p < 0.01, *** - p < 0.001, **** - p < 0.0001 (color-coded asterisks indicating differences versus diagnosis time; black brackets demonstrating essential variations between groups).

In the course of chemotherapy bone marrow VSELs were shown to gradually decrease up until the 12^th^ week of observation (p =<0.0001), with the highest decline within first 15 days of treatment implementation (p = 0.0402). Interestingly, despite initially significant reduction of HSCs at the 15^th^ day of therapy (p = 0.0001), the cells gradually moved towards the opposite direction compared to VSELs leading to values even higher that those reported at admission (**Figure 2A**). Next, patient risk-group stratification revealed that in accordance with pre-treatment higher values of VSELs in IR subjects, that group responded most significantly to therapy implementation (p <0.0001). Similarly, HSCs changes were dominant in the patients from the IR group. However, in accordance with previous paragraph an increase was observed after the 15th day of therapy. In addition, primarily lower HSC levels in HR patients also started to increase, with significantly higher values on the 33^rd^ day (p = 0.0024) (**Figure 2B**).

### 3.3 Association between VSELs and HSCs bone marrow levels at diagnosis and disease-related diagnostic parameters

Considering essential differences in selected stem cells in ALL patients and their change during the course of therapy, we evaluated their relation to the monitored laboratory parameters. Basic assessment of correlations with VSELs and HSCs did not reveal any crucial results, with only tendencies for their moderate positive link to 33^rd^ day leukocyte blood levels (p = 0.077 and p=0.053 accordingly), and hemoglobin level at admission with VSELs separately (p = 0.078) (**Figure 2C**). Furthermore, as higher stem cells predominated in patients with ALL, we aimed at evaluating correlation of different levels of VSELs or HSCs with monitored parameters. We found, inter alia, positive moderate association between higher VSELs and the MRD level (p=0.014) or bone marrow blasts on the 15^th^ day of therapy (p=0.08). Interestingly, strong negative correlation was demonstrated between the ALL group with low level VSELs and peripheral blood leukocytes (WBC) on the 15^th^ day (p=0.007). In contrast to high-VSEL group, positive correlation with day 15 bone marrow blasts was shown in reference to HSCs of low-HSC group (p=0.018). Simultaneously, the same stem cell population demonstrated moderate negative link to peripheral blood blasts levels on the 8^th^ day of chemotherapy (p=0.04). In context of associations at diagnosis, most significant correlations were shown predominantly in high-HSC patients, where HSC were positively associated with bone marrow blasts (p =0.0459) (**Figure 2D**).

### 3.4 Significance of selected stem cell populations in changes of hematological parameters in response to chemotherapy in pediatric ALL management

**Figure 3:**
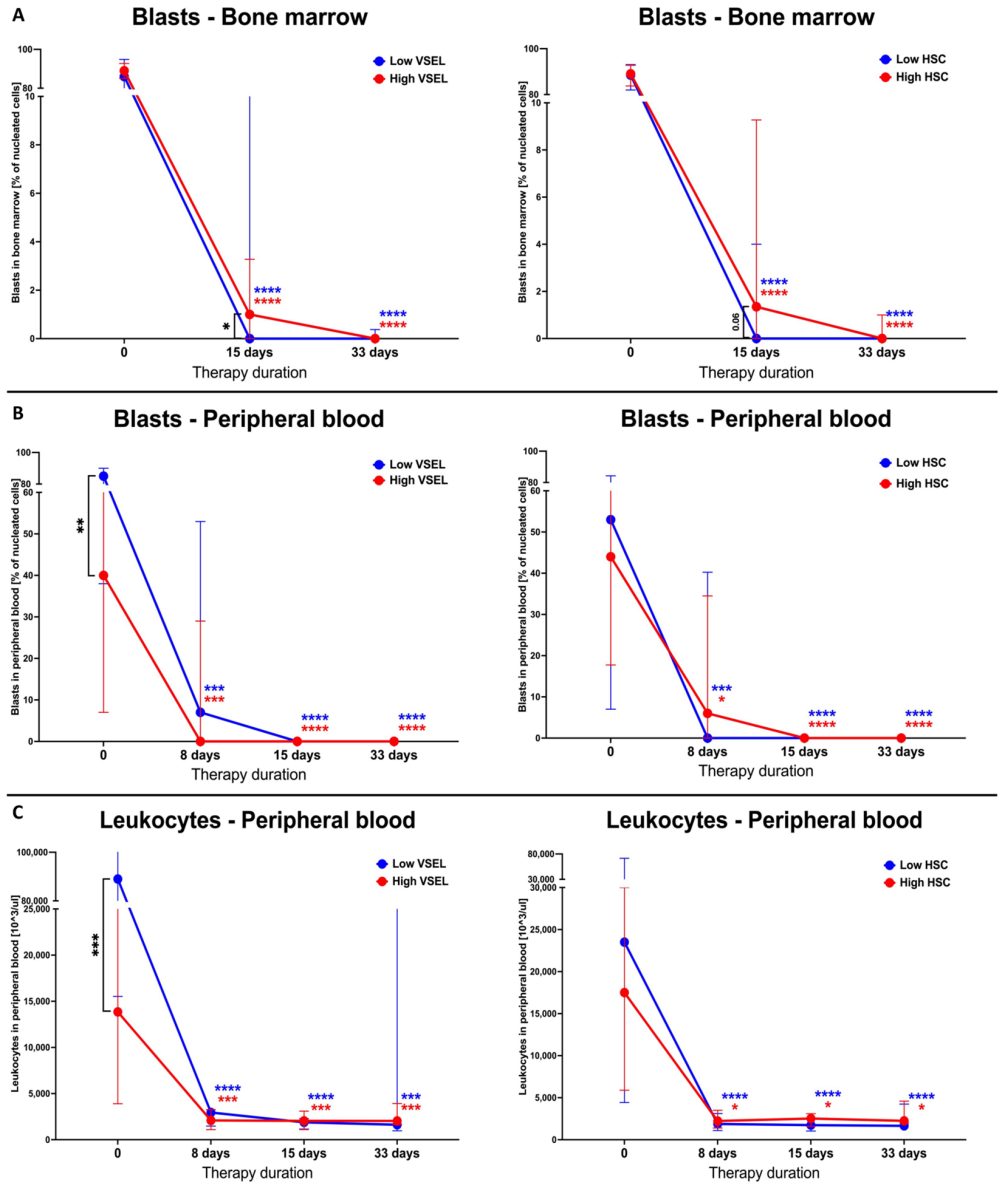
Assessment of the bone marrow various VSEL and HSC levels influence on the response of ALL pediatric patients to the chemotherapy. Stratification of ALL patients on the basis of low/high levels of VSEL or HSC (median-based) and visualization of changes in the bone marrow blasts **(A)**, peripheral blood blasts **(B)** and leukocytes **(C)**, in response to the therapy. Data presented as medians with interquartile range. Statistically significant values were indicated with asterisks: * - p < 0.05, *** - p < 0.001, **** - p < 0.0001 (color-coded asterisks indicating differences versus admission time; black brackets demonstrating essential variations between groups).

Implementation of therapy in ALL patients was associated with highly efficient eradication of blast cells, both in the peripheral blood and bone marrow (p < 0.0001). Noteworthy, we found that different levels of VSELs and HSCs might affect responses to the applied treatment. Despite no initial differences in bone marrow blasts, we demonstrated more efficient reduction of neoplastic cells at 15^th^ day in ALL patients with lower VSELs (p <0.0001) or HSCs (p < 0.0001) (**Figure 3A**). Regarding lymphoblast levels in the peripheral blood cells, this parameter was significantly lower at diagnosis in subjects with increased VSEL levels compared to those patients with lower VSEL count (p = 0.0147). In addition, pediatric ALL patients with lower VSEL levels on the 8^th^ day of therapy seemed to respond comparably worse to treatment (low vs high). In contrast, at the same time point, higher-HSC group did not achieve such effective blast reduction as ALL patients with reduced HSCs (high vs low) (**Figure 3B**). As expected, changes in peripheral blood leukocytes followed those reported in blasts. Here, we did not demonstrate any significant differences in response to the therapy between low and high VSELs or HSCs. At the diagnosis stage, only ALL patients with low VSEL levels were found to have significantly higher leukocyte levels compared to high-VSEL group (p = 0.002) (**Figure 3C**).

### 3.5 Assessment of associations between ALL risk-group stratification and selected stem cells and their contribution to the therapy outcome

**Figure 4:**
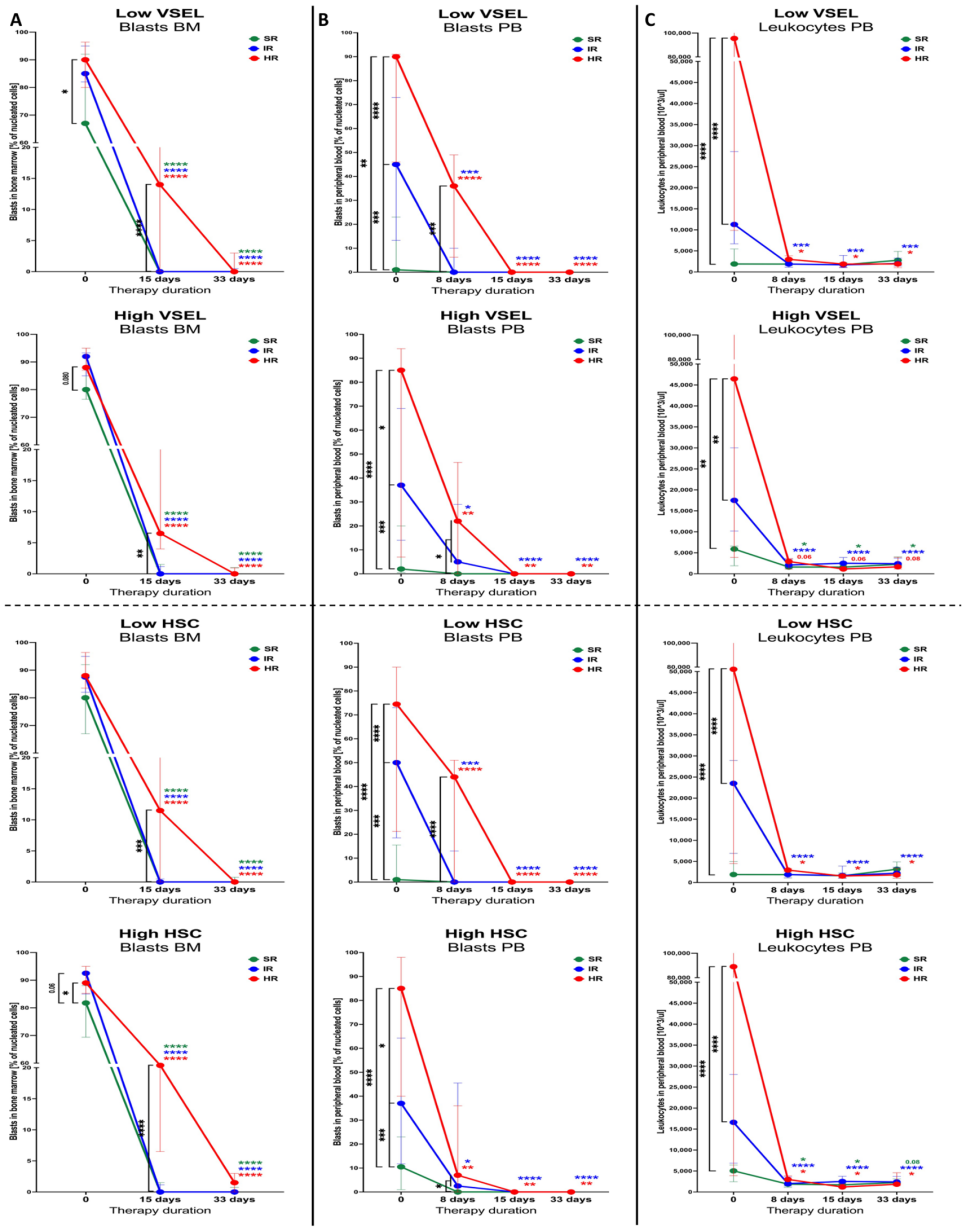
Changes in clinically-relevant laboratory parameters in ALL patients stratified on the basis of the disease outcome prognosis (SR, standard; IR, intermediate; HR, high risk). The therapy affected variations in bone marrow blasts **(A)**, peripheral blood blasts **(B)** and leukocytes in the peripheral blood **(C)** were demonstrated within subjects with low or high VSEL levels, and low or high HSC levels. Data presented as medians with interquartile range. Statistically significant values were indicated with asterisks: * - p < 0.05, ** - p < 0.01, *** - p < 0.001, **** - p < 0.0001 (color-coded asterisks indicating differences versus admission time; black brackets demonstrating essential variations between groups).

Stratifications of ALL patients into prognostic factors-based risk groups has a direct translation into the outcome of the first therapeutic approaches and later stages of management. We examined whether analyzed VSEL and HSC populations affect the response to treatment in the subjects characterized with different initial levels of these stem cells. First, we found that a smaller decline in bone marrow blasts in high-VSEL group at the 15^th^ day was exclusively related to HR patients (p < 0.0001). Interestingly, higher values of VSELs seemed to be associated with comparably more favorable changes in lymphoblasts. Similar phenomenon was observed in reference to high HSCs, with less effective bone marrow blast reduction in ALL subjects from the HR group (p < 0.0001). Here, however, improved decline in blasts was showed when HR patients demonstrated lower HSC levels (**Figure 4A**). Risk-based stratification in peripheral blasts including different VSEL or HSC level showed close similarity to those observed in the bone marrow niche. The only difference was associated with higher HSCs contributing to better responses in HR patients, in contrast to domination of lower HSCs in better bone marrow blasts reduction (**Figure 4B**). Despite additional stratification of the ALL patients into severity groups, no additional significant differences were demonstrated in reference to peripheral blood leukocytes (**Figure 4C**).

### 3.6 Prognostic value of VSEL and HSC populations in monitoring ALL treatment outcome

**Figure 5:**
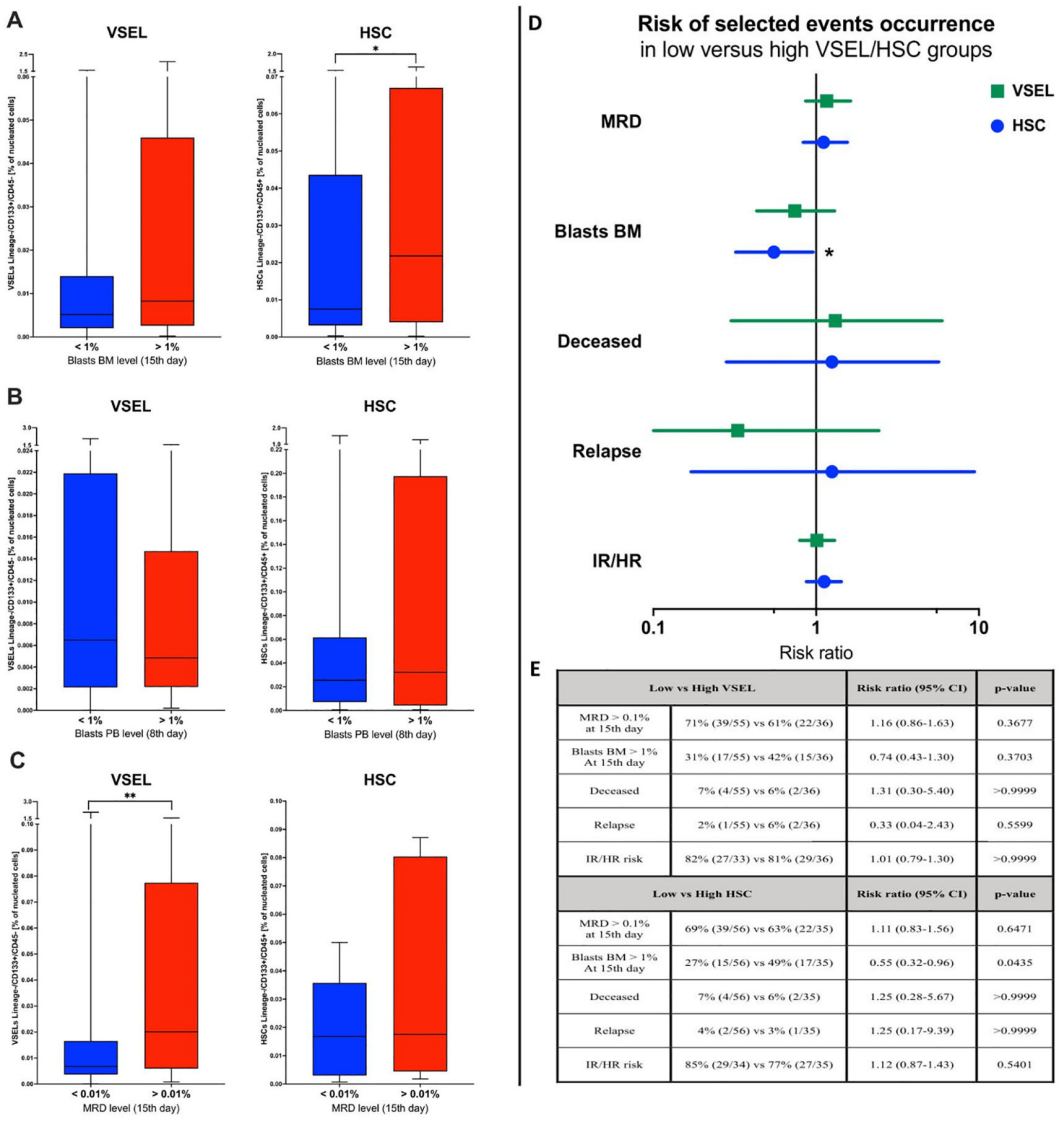
Assessment of VSEL and HSC populations in context of the therapy effectiveness. Differences in selected stem cells between patients with below/above: 1% of bone marrow blasts (at 15^th^ day) **(A)**, 1% of peripheral blood blasts (at 8^th^ day) **(B)**, and 0.01% of MRD level (at 15^th^ day) **(C)**. Evaluation of relative risk of selected events occurrence in context of low/high VSEL of HSC levels reported at admission **(D)**; graphic supported by tabular data **(E)**. Data presented as Turkey boxplots or median values exclusively; risk assessment results presented as risk ratio with 95% confidence intervals. Tabular data included frequency of selected events occurrence (with number of subjects within total group analyzed in brackets). Statistically significant values were indicated with asterisks: * - p < 0.05, ** - p < 0.01.

In accordance with the results above, we decided to verify the value of selected stem cells assessment at the admission stage as prognostic factors of response to the implemented treatment. We did not find any differences in VSELs or HSCs between patients who demonstrated peripheral blood blasts of below or above 1% at the 8^th^ day of therapy (**Figure 5A**). Noteworthy, ALL patients with bone marrow blast above 1% at the 15^th^ day showed significantly higher values of HSCs (p = 0.019) (**Figure 5B**). Interestingly, despite no crucial differences in previous parameters, higher VSEL levels at admission were found in subjects with MRD levels above 0.01% at the 15^th^ day of treatment (p = 0.009) (**Figure 5C**). Considering the fact that initial VSEL and HSC levels might not precisely predict future outcome, we implemented risk assessment analysis. Here, we did not report significant contribution of different selected stem cell levels to patients’ death, disease severity or MRD. Nonetheless, subjects with lower initial VSELs seemed to have reduced risk of the relapse at later therapy stages (RR = 0.33). Moreover, ALL patients with lower HSCs at time of admission showed significantly lower risk of unfavorable bone marrow blast levels (> 1%) at 15^th^ day of therapy (RR = 0.55; p = 0.0435) (**Figures 5D, E**).

### 3.7 Evaluation of the SDF-1 chemokine in context of VSELs and HSCs distribution in ALL patients subjected to the chemotherapy

**Figure 6:**
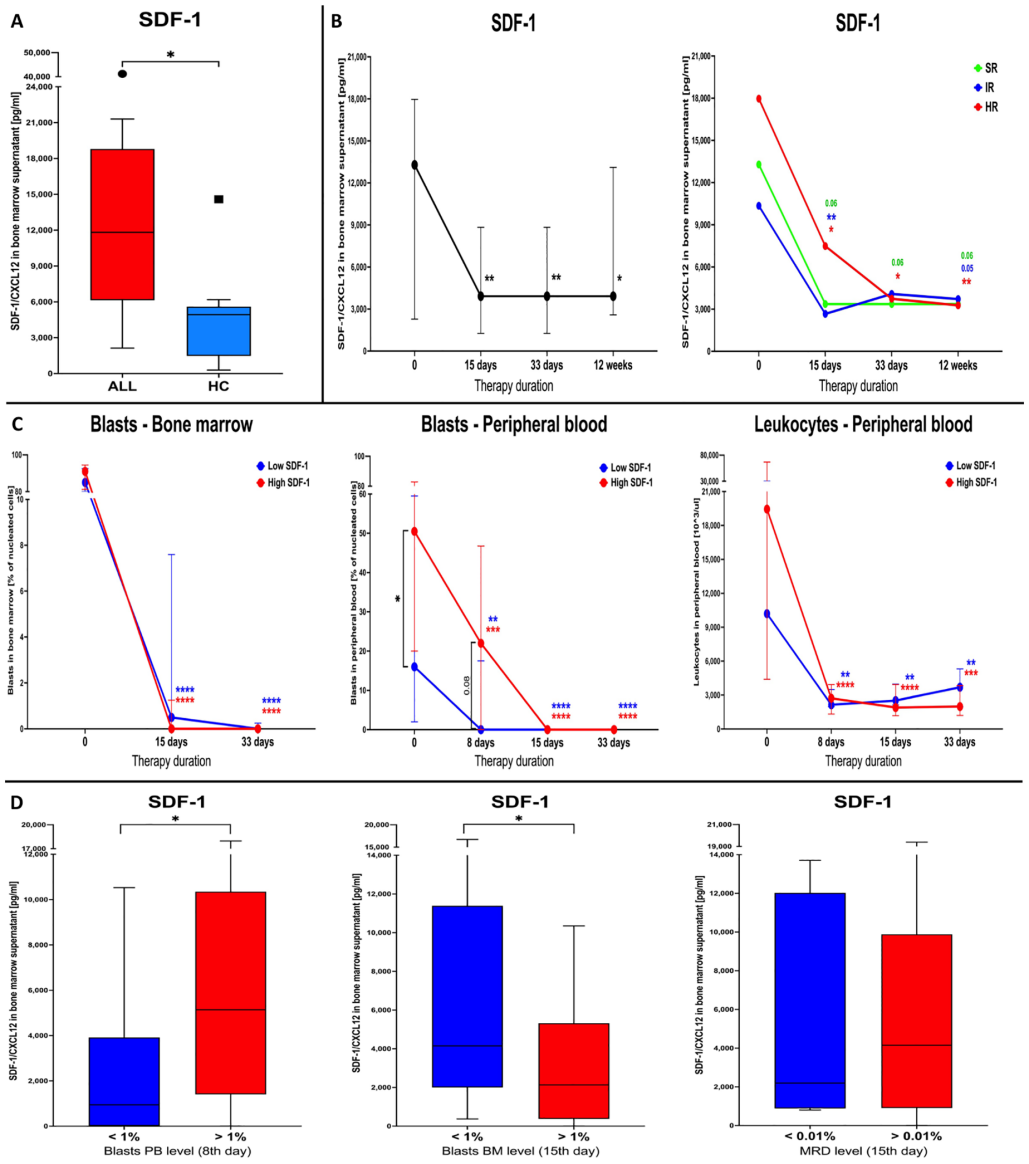
Evaluation of SDF-1 chemokine in ALL patients subjected to chemotherapy. Differences in SDF-1 bone marrow level between ALL patients and healthy control group (HC) at the admission **(A)**. Analysis of changes in SDF-1 response to the therapy in ALL patients, and additionally distinguished by risk-based stratification (SR, standard; IR, intermediate; HR, high risk) **(B)**. Stratification of ALL patients on the basis of low/high levels of SDF-1 (median-based) and visualization of changes in bone marrow blasts, peripheral blood blasts and leukocytes, in response to therapy **(C)**. Differences in the initial SDF-1 concentration between patients with below/above: 1% of peripheral blood blasts (at 8^th^ day), 1% of bone marrow blasts (at 15^th^ day), and 0.01% of MRD level (at 15^th^ day) **(D)**. Data presented as Turkey boxplots, medians with interquartile range or median values exclusively. Statistically significant values were indicated with asterisks: * - p < 0.05, ** - p < 0.01, *** - p < 0.001, **** - p < 0.0001 (color-coded asterisks indicating differences versus admission time; black brackets demonstrating essential variations between groups).

We confirmed that reported high levels of VSEL and HSC populations within the bone marrow of ALL subjects were concomitantly associated with increased concentration of SDF-1 in the niche compared to the control group (p = 0.0185) (**Figure 6A**). Furthermore, such association between selected stem cells and SDF-1 was also shown during therapy implementation, with significant decline in the chemokine level on the 15^th^ day (p = 0.0016) and maintained through the 33^rd^ day (p = 0.0016) until the final 12^th^ week of treatment (p = 0.0178). Despite slightly higher values in HR patients at the admission stage, in general, there were no statistically significant differences in the context of patient risk stratification (**Figure 6B**).

Regarding changes in hematological data during therapy, we found no differences between subjects with low or high bone marrow SDF-1 in reference to the bone marrow blast levels. In addition, that parameter followed the same direction as in patients stratified on the basis of VSELs or HSCs, with significant decrease on the 15^th^ and 33^rd^ day (p < 0.0001). Interestingly, subsequently we found that ALL patients with lower SDF-1 demonstrated significantly lower initial values of peripheral blood blasts (p = 0.0257). Furthermore, the same subjects achieved the blast level median below 1% quicker than high-level SDF-1 patients (p = 0.08) – on the 8^th^ day of the treatment (p = 0.0025). Higher leukocytes in ALL patients were similarly observed in the group of high SDF-1 concentration within the bone marrow. However, despite later significant decline in that parameter, no differences were shown between low and high SDF-1 level groups (**Figure 6C**).

Further evaluation revealed that ALL patients that achieved peripheral blood blasts below 1% on the 8^th^ day the therapy were characterized with significantly lower SDF-1 concentrations in the blood at the admission stage (p = 0.0268). On the contrary, subjects with bone marrow blasts below 1% showed higher levels of SDF-1 chemokine on the 15^th^ day of the patients’ monitoring (p = 0.0464). In reference to MRD, we did not observe any statistically significant differences in SDF-1 between ALL groups with favorable (below 0.01%) or unfavorable level of this hematological parameter on the 15^th^ day of treatment (**Figure 6D**).

## 4 Discussion

Acute lymphoblastic leukemia is characterized by a malignant proliferation of B and T-cell precursors deriving from hematopoietic stem cells in the bone marrow. Despite relatively positive therapy outcome in childhood ALL, there are still reported cases of disease relapse resulting from ineffective disease eradication or alterations of the primary leukemic clone (34). Therefore, there is a need for greater understanding of this malignant hematopoiesis especially in the context of the behavior of stem cells coexisting alongside lymphoblasts in the leukemia niche, considering several hypotheses underlying the role of stem cells, including VSELs in tumorigenesis, possibly through their transformation into cancer stem cells (CSCs) (35, 36). Our study allowed us to establish crucial changes in the two stem cell populations – VSELs and HSCs, with their role in the ALL course. Furthermore, the impact of chemotherapy on these populations was exhibited by the contribution of selected stem cells in the final outcome of ALL management.

At the moment of diagnosis, the bone marrow aspirates of our pediatric ALL patients revealed high levels of not only HSCs (contributing to the development of the myeloid- and lymphoid-lineage cells), but also very small embryonic-like stem cells – VSELs – to date described as exhibiting highly regenerative potential upon activation due to tissue damage inter alia in hepatic injury or limb ischemia (37, 38). Interestingly, we additionally observed a disturbed ratio between these stem cells within the leukemia subjects, with the control group exhibiting significantly lower levels of VSELs compared to HSCs. At this point it is hard to hypothesize whether the changes in stem cell levels are the cause or the result of the neoplastic process. This is especially due to the fact that increase in VSELs or HSCs was not exclusively reported in patients with high disease severity, but rather demonstrated within subjects stratified into the intermediate risk group. Years back, a few reports suggested the participation of stem cells, closely resembling VSELs, in the tumorigenic process including ovarian or testicular cancer (39, 40). Nowadays, however, these aspects are questioned and embryonic-like stem cells role is rather considered positive in context of affected tissue restoration (41).

The analyzed patient group underwent treatment according to the ALL-IC BFM 2009 protocol. The induction phase, on which we mainly focused our observations on, involved receiving both oral glucocorticosteroids – prednisone, intravenous chemotherapeutic agents – vincristine, daunorubicin, L-asparaginase, and intrathecal methotrexate. Noteworthy, the analyzed stem cells responded significantly to the applied regiment. Interestingly, although VSELs declined gradually at subsequent stages of the treatment monitoring with the highest drop on the 15^th^ day, HSCs after the first decrease on the 15^th^ day subsequently started to increase towards values reported at diagnosis. Initial decline in both stem cell populations can be explained by the strong correlation between VSELs and HSCs in ALL patients and the inevitable cytotoxic effect of the applied treatment. However, the phenomenon occurring within HSCs after the 15^th^ day of therapy seems to be associated with the restoration of their cell pool. This could be additionally supported by the concomitant decline in VSELs, most probably reconstituting HSCs. Our data seems to represent a significant step forward in explaining the role of VSELs in the hematopoiesis restoration in the course of acute leukemia management. Previous mice models of ALL indicated the initial drop in the bone marrow total nucleated cells in response to chemotherapeutic agents (5-flurouracil). Concomitantly, in consent with our study, subsequent days indicated reconstitution of bone marrows function with an increase in HSCs, probably through VSEL differentiation, seen predominantly in young subjects (42). Surprisingly, the exact impact of the course of acute lymphoblastic leukemia therapy on the bone marrow niche and the residing stem cells has not been thoroughly studied. A study by Cox et al. revealed that an *in vitro* coculture of normal hematopoietic stem cells with dexamethasone or vincristine left them relatively unaffected in response to the administered therapeutic agents (43). On the other hand, Cetin et al. identified corticosteroids as apoptotic agents in the hematopoietic stem cell differentiation. Interestingly, released hematopoietic cytokines counteracted the steroid effect, by decreasing the level of selected apoptotic markers (44). This dependency indicates that the notable decline in stem cell populations we observed after implementing anti-leukemic treatment is not solely related to the direct drug-cell interaction, but could be more associated with the effect on the bone marrow niche as a whole. Noteworthy, Tang et al. reported that the sympathetic neurotoxicity of vincristine may be responsible for discrepancies in stem cell proliferation leading to the possible evolution to a malignant state (45). In consent with our study, lower level of VSELs was reported in the peripheral blood of adult ALL patients in complete remission after treatment, confirming the dampening effect of ALL treatment on the bone marrow niche including the selected stem cell populations (46).

At further stages of the investigation, we did not find direct statistically significant correlations between selected stem cells – VSELs or HSCs, and hematological data – with only tendencies for positive moderate association between both populations and 33^rd^ day peripheral blood leukocyte levels. Interestingly, when dividing patients into low/high level stem cell groups, those with high HSCs showed positive correlation with bone marrow blasts at diagnosis. In addition, in lower or higher VSELs group, those stem cells correlated moderately positive with the 33^rd^ day bone marrow blasts or the 15^th^ day MRD respectively. Similarly, HSCs also correlated with bone marrow blasts on the 15^th^ day of therapy. Those results might be partially associated with high proliferative feature of both stem cell populations. In mice model of ALL hematopoietic stem cells such property was related to uncontrolled expansion and higher risk of leukemia occurrence (42). Moreover, VSELs in low-VSEL ALL patients showed strong negative correlations with leukocytes on the 15^th^ day, and this could indirectly indicate relationship to the restoration of the HSCs pool – where decline of VSELs leads to the development of HSCs that further give rise to the new leukocytes pool. In fact, the study of hematopoiesis in the course of pregnancy (mice model) highlights the indirect role of VSELs in the process, with concomitant role in the development of other organs. Demonstrated co-expression of NUMB (cell fate determinant during development) and OCT-4 (pluripotency marker) proteins in VSELs supported the significant role of those stem cells in generation of multipotent HSCs responsible for actual regeneration of the hematopoietic cells pool (47).

Considering the information above, we intended to verify whether initial level of VSELs or HSCs has any contribution to the changes in crucial hematological parameters in response to the chemotherapy implementation. Most importantly, patients with lower VSELs or HSCs had slightly improved response to the applied therapy reflected by a better reduction of the bone marrow blast levels measured on the 15^th^ day. In the end, however, all tested subjects reached the same efficiency in neoplastic cell reduction. No significant differences in response to the therapy were observed in reference to the peripheral blood blasts or leukocytes. Nevertheless, those ALL patients with lower VSEL levels demonstrated substantially higher leukocytosis and peripheral blasts before commencement of therapy. High values of the leukemia-related parameters in those subjects with reduced VSELs are a substantial complement to the reports on the indirect influence of VSELs on hematopoiesis (47). We were able to further support that hypothesis when additionally stratifying patients into risk groups and monitoring blast and leukocyte levels. Retrospective analysis allowed us to evaluate the differences between low/high VSEL or HSC groups and risk-groups assigned to the patients according to the ALL-IC BFM 2009 protocol. Regarding the level of bone marrow blasts, we showed that higher initial values were observed in patients with lower VSELs or higher HSCs in the HR group. Noteworthy, the same HR patients – from low VSELs or high HSCs group, demonstrated worse response to the implemented therapy than other risk-based groups, and also opposing HR groups in context of stem cell levels. Similar changes were reported in the context of peripheral blood blasts, with the exception of the lower HSCs HR patients demonstrating reduced efficacy of ALL therapy. We presume that apart from demonstrated differences in values of VSELs or HSCs, some qualitative properties might be responsible for response to the therapy in studied ALL risk groups. Interactions between stromal and leukemic cells were found to be affected by CD9 (tetraspanin 29, TSPAN-29) in myeloid cancers (48, 49). VSEL-like cells isolated from a myeloproliferative neoplasm showed significantly higher expression of that protein, with higher proliferative and chemotactic potential of CD9+ stem cells (50). In accordance, the role of selected proteins combined with data presented in our study provides novel insight into cooperation between VSELs and HSCs in the hematopoietic process.

Although subtle, variations in treatment response of ALL patients in the context of the stem cell levels encouraged us to investigate the potential prognostic value of the VSELs and HSCs. We found, inter alia, that pediatric patients who responded well to the therapy and achieved bone marrow blasts below 1% on the 15^th^ day of the induction phase of the treatment had significantly lower HSCs levels at diagnosis. Moreover, subjects that responded more favorably in the context of MRD measured on the 15^th^ day had substantially lower VSEL values before treatment implementation. In fact, in the risk analysis we indicated that initial higher HSCs are associated with a risk of unfavorable blast level on day 15 in the bone marrow (> 1%) almost twice as high as compared to those with low HSCs before therapy. Importantly, despite the lack of statistical significance low-VSEL subjects seemed to have three times less risk of relapse than the high-VSEL patient group. As mentioned above, significantly lower levels of VSELs were indeed demonstrated in ALL patients from the lower/intermediate risk of poor response to therapy. Interestingly, our study provides next substantial support for diagnostic value of stem cell monitoring in therapy, indicated recently by Bhartiya et al. as potential candidates in cancer monitoring (51).

Lastly, we intended to reveal whether changes in VSELs and HSCs are somehow related to one of their most crucial chemoattractant – stromal cell-derived factor 1 (SDF-1; CXCL12), associated especially with their mobilization - migration into periphery and homing into tissues process (52). As expected, we reported initially high SDF-1 concentrations in the bone marrow at admission stage. Variations in this chemokine closely reflected changes in VSEL levels, with their concomitant significant reduction in the course of therapy. That is an important, clinical complementation to previously demonstrated *in vitro* close relation of SDF-1 levels to VSELs expressing the CD9 marker (50). Comparable, yet less pronounced, link between tested stem cells, both HSCs and VSELs, was demonstrated in the course of a septic shock. Surprisingly, another stem cells chemoattractant – S1P (sphingosine-1-phosphatate), was significantly reduced in those patients (53). Furthermore, we found quite opposite effects of the low/high SDF-1 levels compared to the VSEL-based stratification. Here, higher SDF-1 levels were associated with initially higher peripheral blood blasts, which is in accordance with results published by Nishii et al. who found SDF-1 to promote lymphoblast survival (54). However, no other differences were reported, and as in stem cells, further observation showed the same efficiency of therapy for all studied groups. Finally, we showed that better response to therapy with reduction of blasts in peripheral blood below 1% is associated with lower initial SDF-1 concentrations in the bone marrow. On the contrary, higher SDF-1 at admission stage was later linked to a higher decline in the bone marrow lymphoblasts. Importantly, despite significant role of the SDF-1 in the ALL-related changes in the stem cells pool, we must remember that other well-known factors can also influence observed phenomenon: S1P, ATP, C1P, follicle stimulating hormone (FSH) – promoting, or CD39 and CD73 – inhibiting (through extracellular adenosine) mobilization of the stem cells (55–57). In accordance, further studies could be extended with mechanistic verification of the pathways playing a dominant role in the course of leukemia, including acute lymphoblastic leukemia.

Studied stem cells, namely VSELs and HSCs, were found to be crucial participants in the phenomenon occurring in the course of pediatric ALL therapy. These populations responded to the implemented treatment in the way initially suggesting their participation in the neoplastic process. Interestingly the observed restoration of the new leukocytes’ pool, with VSELs most probably contributing to an increase in HSCs later in therapy, speaks in favor of their beneficial role. Selected stem cells did not allow us to distinguish groups with better or worse response to the induction phase of therapy as the applied treatment was highly effective in all patients in this phase. However, slight early-stage differences in VSELs or HSCs might be helpful in predicting more or less favorable responses within first two weeks of treatment and, thus, indicate more caution towards monitoring of these subjects. Tested populations of cells should be used together as predictors of the therapy outcome because each of them demonstrated different values in monitoring in context of later blast or MRD levels. Further investigation of VSELs and HSCs in the clinical practice might allow for verification of their potential as biomarkers of the disease outcome. Moreover, increasing the patient groups would be essential in establishing whether these subsets of cells could be beneficial in clearer identification of those pediatric ALL patients with higher risk of worse outcome already at the admission stage.

## Data Availability

The raw data supporting the conclusions of this article will be made available by the authors, without undue reservation.
