# Supplementary material for: Selected stem cell populations in pediatric acute lymphoblastic leukemia
Source: Front Immunol. 2024 Sep 25;15:1446687. doi: 10.3389/fimmu.2024.1446687 (PMC11461207; doi:10.3389/fimmu.2024.1446687)
Supplement: Supplementary file 1 [file DataSheet1.pdf]

Supplementary Figures

A

ALL pediatric patients	
**Subjects number**	60
**Sex distribution** [female vs male]	40% vs 60%
**Age** [years]	5.0 [3.0; 10.5]
**Weight** [kg]	18.05 [16.63; 34.63]
**Height** [cm]	110.5 [98.0; 138.5]
**Leukocytes** [10^3/µl]	15.80 [4.98; 36.86]
**Blasts peripheral blood** [% of nucleated cells]	46.50 [14.50; 78.50]
**Blasts bone marrow** [% of nucleated cells]	88.00 [80.00; 93.08]
**Platelets (PLT)** [10^3/µl]	51.50 [18.00; 122.28]
**Hemoglobin (Hb)** [g/dl]	7.750 [6.35; 9.85]

B

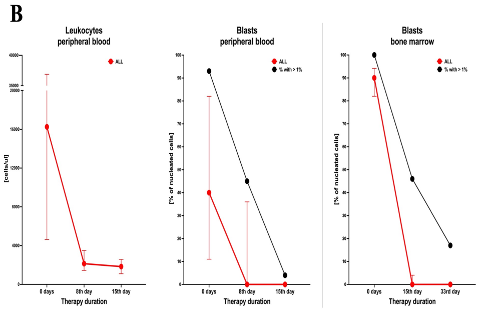

Monitoring parameters of treated ALL pediatric patients				
	Diagnosis (day 0)	8th day	15th day	33rd day
**Leukocytes** [10^3/µl]	15.80 [4.98; 36.88]	2.17 [1.41; 3.42]	1.67 [1.10; 2.58]	2.02 [1.64; 3.58]
**Blasts peripheral blood** [% of nucleated cells]	46.50 [14.50; 78.50]	0.00 [0.00; 28.75]	0.00 [0.00;0.00]	0.00 [0.00;0.00]
**Blasts bone marrow** [% of nucleated cells]	88.00 [80.00; 93.08]	-	0.00 [0.00; 4.00]	0.00 [0.00;0.00]

C

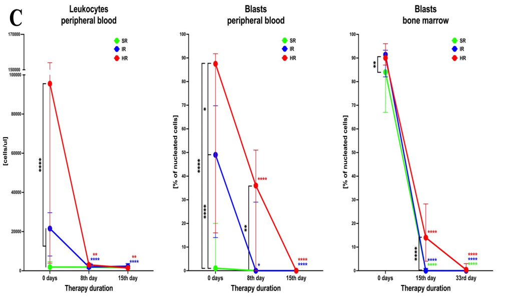

	Treatment response in different ALL risk groups				
	Risk group	Diagnosis (day 0)	8th day	15th day	33rd day
**Leukocytes** [10^3/µl]	SR	4.19 [1.99;6.43]	1.61 [1.19;2.21]	1.60 [1.35;2.10]	2.40 [1.75;3.85]
	IR	19.25 [7.06; 29.65]	2.01 [1.33; 3.63]	2.31 [1.01; 3.70]	2.21 [1.97; 3.67]
	HR	67.00 [5.98; 192.00]	2.97 [2.29; 3.65]	1.19 [1.10; 1.90]	1.97 [1.00; 2.99]
**Blasts peripheral blood** [% of nucleated cells]	SR	11.00 [1.00; 61.50]	0.00 [0.00; 0.75]	0.00 [0.00;0.00]	0.00 [0.00;0.00]
	IR	46.50 [15.50; 69.75]	0.00 [0.00;26.50]	0.00 [0.00;0.00]	0.00 [0.00;0.00]
	HR	74.50 [27.25;95.25]	29.00 [0.00;46.00]	0.00 [0.00;0.00]	0.00 [0.00;0.00]
**Blasts bone marrow** [% of nucleated cells]	SR	80.00 [67.00; 88.00]	-	0.00 [0.00; 1.13]	0.00 [0.00;0.00]
	IR	92.00 [85.00; 95.00]	-	0.00 [0.00; 1.00]	0.00 [0.00;0.00]
	HR	87.50 [65.00; 93.75]	-	11.50 [1.00; 32.75]	0.00 [0.00; 0.75]

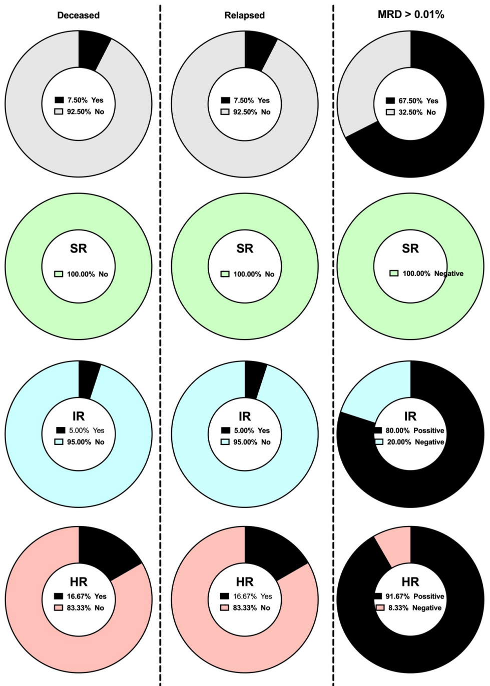

Supp. Fig. 1. Characteristics of ALL patients enrolled in the study. Basic anthropomorphology and hematology of studied subjects at admission (A). Changes in peripheral blood/ bone marrow blasts and blood leukocytes in the course of therapy (black lines in blast data indicate additionally percentage of patients with specific level of the cells) (B). Visual and tabular

presentation of treatment influence on hematological parameters of the patients stratified on the basis of risk group (SR – standard/ IR – intermediate/ HR – high risk) (C). Distribution of deceased, relapsed or demonstrating > 0.01% MRD level ALL patients, including risk stratification. Tabular or graphical data presented as medians with interquartile range Statistically significant values on graphs were indicated with asterisks: * - p < 0.05, ** - p < 0.01, *** - p < 0.001, **** - p < 0.0001.

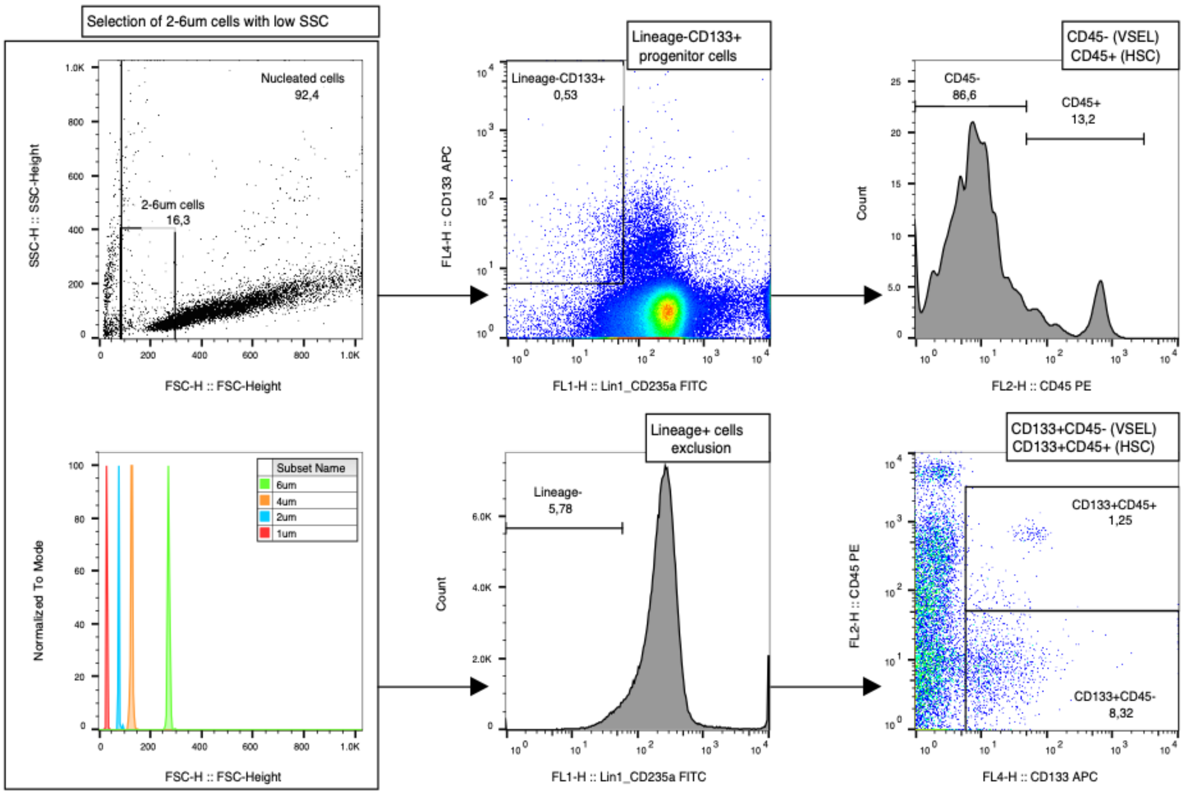

Supp. Fig. 2. Gating strategy for selected stem cell populations immunophenotyping. VSEL, HSC were distinguished on the basis of morphology and size of 2-6µm, absence of mature leukocyte markers (Lineage1: CD3, CD14, CD16, CD19, CD20, CD56) and erythrocytes (CD235a), presence of CD133 and differential expression of CD45. In accordance, Lin1-CD133+CD45+ (HSC) and Lin1-CD133+CD45- (VSEL) were distinguished. Bidirectional gating was used for technical verification of the analysis technique.
